# Supplementary material for: Shared autonomic phenotype of long COVID and myalgic encephalomyelitis/chronic fatigue syndrome
Source: PLoS One. 2026 Jan 23;21(1):e0341278. doi: 10.1371/journal.pone.0341278 (PMC12829881; doi:10.1371/journal.pone.0341278)
Supplement: Attachment — Submitted filename: Peer Review PONE - D - 25 - 02329.pdf [file pone.0341278.s001.pdf]

Recommendation - Major Revision

Introduction

1. Recommend in the 1st few lines explaining how they “disable several organ systems” or give a one line explanation of the common symptoms and disabilities patients experience
2. Highlight what similarities are seen early in the introduction. A large portion of the beginning keeps referring to symptoms without enough background information
3. Missing references for the line starting with “ Both disorders..”, “They are speculated” and “Both disorders are believed to be…”
4. Missing references for “About 25%...”
5. The line “Different elements..” needs to be rephrased - Remains too lengthy with multiple “and” conjunction in sentence structure
6. Recommend adding why hEDS patients were chosen as part of the control group or at least the clinical reasoning behind this addition of patients.
7. Recommend adding what clinical question and hypothesis was aimed to be ascertained in the study. Needs more clarification

Impression - Needs further background information of what was done in prior relationships and what other researchers had discovered about this phenom. I would also recommend more background information of why the comprehensive testing was selected in this way and explain deeper what the clinical question and purpose of the study. The introduction is written in a way whereby the onus remains on the reader to have a reasonable understanding of this topic to fully grasp the information which reduces the impact of the information following.

Materials and Methods

1. Recommend rephrasing the line starting with “ Hypocapnic cerebral hypoperfusion..”.
2. Recommend rephrasing “ Orthostatic cerebral hypoperfusion…”. This line also has a closing bracket incorrectly
3. Why was inclusion and exclusion criteria not bolded with the rest of the paragraph separators
4. There was no statement about what age group was used in the inclusion/exclusion
5. Recommend removing the “We” from “We followed the…”
6. Adding the criterias for diagnosis would aid the reader to understand how the patients were decided upon
7. How were healthy controls chosen before testing them?
8. Recommend re - doing the QASAT paragraph into a more reading friendly manner.

Impression - Gaps noted in necessary parts of the materials and methods. Difficult to read and ascertain the important information. Recommendation to reduce the verbosity in certain key areas, summarize, rephrase and present the information in a simpler way to communicate the definitions and especially the QASAT paragraph better.

Statistical Analysis

1. Missing power analysis. What calculations were done to ascertain the study population needed for significant values
2. What variables were continuous or categorical. Were values needed to be reorganized in different categories?
3. How was the data cleaned or pre processed? What was done for outliers
4. The study does not specify whether normality and homogeneity of variance assumptions were checked before performing ANOVA
5. No reported random effects structure
6. No discussion of interaction effects (Group × Position). This would clarify whether different patient groups had distinct responses to tilt.
7. What adjustments were made for possible confounders?
8. Missing sensitivity analysis to assess impact of missing and ignoring data

Impression - There is a surprising lack of crucial data to understand if the figures about to be presented in the result section are believable given the large gaps in the analysis. At this point the results can be heavily scrutinized and would completely undermine the study as the readers do not have enough structure to accept the figures.
While the basic analysis was explained, the details of the analysis and imputation methods for robustness checks of the data itself remain crucial to accept the results with expected gravitas. This would remain the most important of the study and heavily weighs on the Major revision recommendation

Results

1. This is the 1st time the study mentions how many patients were studied
2. Why was the differentiation of long covid patients based on virus type only mentioned now? Recommend mentioning before in the Materials and methods as well.
3. How was the differentiation of the viruses important then? This also was not mentioned in the statistical analysis
4. What symptoms were shorter duration?
5. None of the 2nd paragraph makes sense. Why does it matter if the hEDS had more irritable bowel syndrome or mast cell activation syndrome? How does all of this fit in?
6. We were not presented on the characteristics of patients so mentioning BMI, Fibromyalgia etc appear at random. What is the purpose of this now?
7. What does the use of pressor medications mean? Are we discussing midodrine? Fludrocortisone? This needs to be clarified.
8. Is there a need for a separate paragraph for iCPET as this was more historical data and a small portion of the patients had this data available?

Impression - A Difficult read overall with lacking of placement of the data and tables within the body of the results rather than in the appendix. Confusing presentation of random patient factors and data as well in the beginning. Recommend redoing the result sections and include the

graphs rather than rely solely on paragraph presentation. I do believe there are too many acronyms to remember in this paper and recommend not relying on them to deliver the data, it becomes very burdensome to read.
No confidence intervals were shown in the data to determine how much variability was noticed.

Discussion

1.
2. Regarding reduced cerebral blood flow, Given the data or lack thereof, did patients with further reduction in CBFv have different or worse symptoms than others?
3. Did they experience more brain fog and was there a discussion to perform standardized cognitive tests to ascertain that relationship? This appears to be a big miss only to detect the reduction in flow and not have it impact for a clinical connection?
4. A note for the longitudinal aspect of ME/CFS vs Long covid however was there a difference in patients by their strain? Was there a difference with patients who had long covid for a prolonged period than other patients?
5. Including a limitation in the middle of the discussion is not warranted. Recommend removing that paragraph and summarizing in the end with the rest of the others.
6. What markers are noted? This was not mentioned before apart from CRP and Interleukin 6
7. How were the markers collected? Was this mentioned in the methods?
8. Multiple missing references noted
9. Typographical error in limitations “a large number many patients”

Impression - Overall a disappointing discussion lacking actual true analysis and speculation on the results found. A noticeable part of it was discussing the studies against the findings for orthostatic hypotension and other parts not fully described e.g. the biopsies done. There was no acceptable differentiation of the different phenotypes of where they differed. It appears the data was collected, not properly presented or explained and just laid out instead of interpreting the data to explain what differences, similarities between the phenotypes and how it relates to a clinical picture. Much of the focus remains on trying to convince the reader the similarities without actually explaining the data. It was striking how little was relayed about the results of the patients and consistently comparing with the different groups especially the healthy control group as I believe they were almost never mentioned. Much more details and deliberation would be needed for this study to make clinical sense as currently it is poorly detailed statistical analysis to detect certain anomalies in these patients then trying to find similarities without having the data itself show the similarities and differences.
