# Supplementary material for: Shared autonomic phenotype of long COVID and myalgic encephalomyelitis/chronic fatigue syndrome
Source: PLoS One. 2026 Jan 23;21(1):e0341278. doi: 10.1371/journal.pone.0341278 (PMC12829881; doi:10.1371/journal.pone.0341278)
Supplement: Attachment — Submitted filename: Response to Reviewers-Final.docx [file pone.0341278.s002.docx]

Dear Editor

We would like to express our sincere gratitude to both reviewers for their constructive feedback. We have carefully revised the paper in accordance with the suggestions. Below, we provide detailed responses to each of the reviewers' comments:

For authors

Peter Novak

Recommendation - Major Revision

Introduction

1. Recommend in the 1st few lines explaining how they “disable several organ systems” or give a one line explanation of the common symptoms and disabilities patients experience

**Answer: Common symptoms associated with both conditions have been added to the introduction**

2. Highlight what similarities are seen early in the introduction. A large portion of the beginning keeps referring to symptoms without enough background information

**Answer: Most of the symptoms are shared in both conditions, that was now clarified in the introduction.**

3. Missing references for the line starting with “ Both disorders.”, “They are speculated” and “Both disorders are believed to be…”

**Answer: We added missing references**

4. Missing references for “About 25%...”

**Answer: We added missing references,**

5. The line “Different elements..” needs to be rephrased - Remains too lengthy with multiple “and” conjunction in sentence structure

**Answer: The sentence was rewritten**

6. Recommend adding why hEDS patients were chosen as part of the control group or at least the clinical reasoning behind this addition of patients.

**Answer: hEDS is a heritable disorder associated with dysautonomia.**

**Therefore, we aimed to assess whether the observed findings represent a common physiological response to illness or if they are specific to Long COVID and ME/CFS. This was added to the introduction.**

7. Recommend adding what clinical question and hypothesis was aimed to be ascertained in the study. Needs more clarification

**Answer: The tested hypothesis was added to the introduction.**

Impression - Needs further background information of what was done in prior relationships and what other researchers had discovered about this phenom. I would also recommend more background information of why the comprehensive testing was selected in this way and explain deeper what the clinical question and purpose of the study. The introduction is written in a way whereby the onus remains on the reader to have a reasonable understanding of this topic to fully grasp the information which reduces the impact of the information following.

**Answer: The introduction was expanded as outlined above.;**

Materials and Methods

1. Recommend rephrasing the line starting with “ Hypocapnic cerebral hypoperfusion**..”.**

**Answer: The sentence was revised.**

2. Recommend rephrasing “ Orthostatic cerebral hypoperfusion…”. This line also has a closing bracket incorrectly

**Answer: The closing bracket was corrected**

3. Why was inclusion and exclusion criteria not bolded with the rest of the paragraph separators

**Answer: The wrong bolding was corrected.**

4. There was no statement about what age group was used in the inclusion/exclusion

**Answer: The inclusion criterion includes the age 18 or older. This was inserted in the methods.**

5. Recommend removing the “We” from “We followed the…”

**Answer: The Sentence was revised.**

6. Adding the criterias for diagnosis would aid the reader to understand how the patients were decided upon

**Answer: The diagnostic criteria for the studied diagnoses was expanded.**

7. How were healthy controls chosen before testing them?

**Answer:**

8. Recommend re - doing the QASAT paragraph into a more reading friendly manner. ?

**Answer: The paragraph was rewritten to make it easier to read. The ranges were move to the tables.**

Impression - Gaps noted in necessary parts of the materials and methods. Difficult to read and ascertain the important information. Recommendation to reduce the verbosity in certain key areas, summarize, rephrase and present the information in a simpler way to communicate the definitions and especially the QASAT paragraph better.

Statistical Analysis

1. Missing power analysis. What calculations were done to ascertain the study population needed for significant values

**Answer: Power analysis was added to the methods.**

2. What variables were continuous or categorical. Were values needed to be reorganized in different categories?

**Answer: Most of variables were continuous. Variables assigned as % and without standard deviation in the bracket (for example percentage of abnormal findings) were categorical.**

3. How was the data cleaned or pre processed? What was done for outliers

**Answer: We did not remove outliers during data cleaning, as they often reflect clinically significant abnormalities rather than errors. From a statistical perspective, these values may appear as outliers; however, in a clinical context, they frequently represent real and meaningful abnormal results. Therefore, we retained these values to preserve the integrity of the clinical information. This decision is further discussed in the Methods section.**

4. The study does not specify whether normality and homogeneity of variance assumptions were checked before performing ANOVA

**Answer: For the continuous variables, we assessed the assumptions of normality and homogeneity of variance. Many of the variables showed non-normal distributions, and there was often a lack of homogeneity of variance between groups. Therefore, instead of using ANOVA, we recalculated data using the Kruskal-Wallis test, which is a non-parametric alternative that does not assume normal distribution and is robust to heterogeneity of variances across groups.**

5. No reported random effects structure

**Answer: This study did not include a random effects structure, as the design did not involve repeated measures or hierarchical/nested data that would require modeling random variability across subjects or groups. All analyses were conducted under the assumption of independent observations. If future studies involve multi-level or longitudinal data, incorporating random effects would be appropriate**

6. No discussion of interaction effects (Group × Position). This would clarify whether different patient groups had distinct responses to tilt.

**Answer: We added the results of the linear model to the results section and also we are discussing the overall difference between groups in the discussion.**

7. What adjustments were made for possible confounders?

**Answer: Yes, the calculations using linear models were adjusted for age and gender as confounders. We expanded the description of how the linear model was used.**

8. Missing sensitivity analysis to assess impact of missing and ignoring data

**Answer: The proportion of missing data was relatively low for both the survey data and autonomic testing, and was therefore deemed unlikely to bias the main findings. However, there were substantial missing values in the laboratory blood work, likely due to test ordering being at the discretion of the attending physicians.**

**To assess the potential impact of missing data on the results, we conducted a sensitivity analysis under the assumption that the data were missing at random (MAR). Specifically, we compared the results of the Kruskal–Wallis test for continuous variables across three scenarios: 11 Complete-case analysis (excluding missing values), 2. Mean imputation (replacing missing values with the overall mean), and 3. Median imputation (replacing missing values with the overall median).**

**The findings remained consistent across all three methods, suggesting that the missing values did not significantly affect the robustness of our results. Details of this analysis have been added to the Methods section.**

**The sensitivity analysis showed that missing values did not significantly affect the robustness of our results.**

Impression - There is a surprising lack of crucial data to understand if the figures about to be presented in the result section are believable given the large gaps in the analysis. At this point the results can be heavily scrutinized and would completely undermine the study as the readers do not have enough structure to accept the figures.

While the basic analysis was explained, the details of the analysis and imputation methods for robustness checks of the data itself remain crucial to accept the results with expected gravitas. This would remain the most important of the study and heavily weighs on the Major revision recommendation

A**nswer: The details of statistical analysis have been added as outline above.**

Results

1. This is the 1st time the study mentions how many patients were studied

**Answer: We added a flow diagram to clarify the number of studied patients.**

2. Why was the differentiation of long covid patients based on virus type only mentioned now? Recommend mentioning before in the Materials and methods as well.

**Answer: We mention the virus strains (pre-Delta, Delta and Omicron) in the method section.**

3. How was the differentiation of the viruses important then? This also was not mentioned in the statistical analysis

**Answer: We had only a small number of patients from the pre-Delta (n=6) and Delta (n=10) periods, as noted in the Results section. Therefore, comparisons between these viral strains are not meaningful due to the limited sample size.**

4. What symptoms were shorter duration?

**Answer: Regarding symptom duration, we asked patients to report the length of time since disease onset or since symptoms first appeared. However, we did not specifically categorize individual symptoms by duration in this study.**

**To clarify this, we added “…**symptom duration defined as the length of time since disease onset.” To the results section and to the table 1.

5. None of the 2nd paragraph makes sense. Why does it matter if the hEDS had more irritable bowel syndrome or mast cell activation syndrome? How does all of this fit in? **Answer: Ehlers-Danlos syndrome (EDS) is associated with a variety of allergic complications, including mast cell activation, as well as multiple pain syndromes and gastrointestinal problems such as irritable bowel syndrome (IBS).**

Hakim A, De Wandele I, O’Callaghan C, Pocinki A, Rowe P. Chronic fatigue in Ehlers-Danlos syndrome-Hypermobile type. Am J Med Genet C Semin Med Genet. 2017;175: 175–180. doi:10.1002/ajmg.c.31542

Gensemer C, Burks R, Kautz S, Judge DP, Lavallee M, Norris RA. Hypermobile Ehlers-Danlos syndromes: Complex phenotypes, challenging diagnoses, and poorly understood causes. Dev Dyn Off Publ Am Assoc Anat. 2021;250: 318–344. doi:10.1002/dvdy.220

6. We were not presented on the characteristics of patients so mentioning BMI, Fibromyalgia etc appear at random.

**Answer: BMI was included as a standard demographic variable. The diagnosis of fibromyalgia is commonly seen in various pain syndromes, including ME/CFS, autonomic dysfunction, and especially small fiber neuropathy. Some studies suggest that patients diagnosed with fibromyalgia may, in fact, have underlying small fiber neuropathy. Given that a significant portion of our study population was on pain medications, we chose to retain the fibromyalgia diagnosis, as it aligns with their clinical presentation and treatment patterns.**

7. What does the use of pressor medications mean? Are we discussing midodrine? Fludrocortisone? This needs to be clarified.

**Answers: The types of pressor medications were clarified in the Table 1 legend. These medications are proamatine, fludrocortisone, pyridostigmine, and droxidopa**

8. Is there a need for a separate paragraph for iCPET as this was more historical data and a small portion of the patients had this data available?

**Answer: Although the number of patients undergoing iCPET was small, the results are valuable because iCPET is an invasive test that provides direct measurements of stroke volume, cardiac filling pressures, and other hemodynamic parameters. These unique findings support and confirm our non-invasive results.**

Impression - A Difficult read overall with lacking of placement of the data and tables within the body of the results rather than in the appendix. Confusing presentation of random patient factors and data as well in the beginning. Recommend redoing the result sections and include the

graphs rather than rely solely on paragraph presentation. I do believe there are too many acronyms to remember in this paper and recommend not relying on them to deliver the data, it becomes very burdensome to read.

No confidence intervals were shown in the data to determine how much variability was noticed.

Discussion

1

2. Regarding reduced cerebral blood flow, Given the data or lack thereof, did patients with further reduction in CBFv have different or worse symptoms than others?

Did they experience more brain fog and was there a discussion to perform standardized cognitive tests to ascertain that relationship? This appears to be a big miss only to detect the reduction in flow and not have it impact for a clinical connection?

**Answer: These are important and insightful questions. While we did not conduct standardized cognitive testing in this particular study, there is a substantial body of literature showing that cognitive function is impaired in both Long COVID and ME/CFS. We agree that establishing a direct clinical connection between reduced cerebral blood flow velocity (CBFv) and cognitive performance would significantly strengthen the findings. In fact, in a recent publication from our group, we demonstrated that reductions in cerebral blood flow correlated with symptom severity in the short term—suggesting that reduced CBF may indeed be related to cognitive decline. However, the absence of standardized cognitive assessments in this study is a limitation. Future research would benefit from incorporating such testing to directly correlate objective cognitive measures with cerebral blood flow metrics. This would help clarify the clinical implications of reduced CBFv, particularly regarding symptoms like brain fog and cognitive dysfunction.**

2. A note for the longitudinal aspect of ME/CFS vs Long covid however was there a difference in patients by their strain? Was there a difference with patients who had long covid for a prolonged period than other patients?

**Answer: That is interesting questions. Due to the small number of pre-Omicron cases, we did not perform a comparative analysis of the effects of different SARS-CoV-2 strains. This is mentioned in the discussion.**

3. Including a limitation in the middle of the discussion is not warranted. Recommend removing that paragraph and summarizing in the end with the rest of the others.

**Answer: The paragraph was moved to the limitation section.**

4. What markers are noted? This was not mentioned before apart from CRP and Interleukin 6

**Answer: The details if inflammatory markers were inserted in the methods.**

5. How were the markers collected? Was this mentioned in the methods?

**Answer: The markers were collected as a of routine clinical evaluations. This is mentioned in the methods.**

6. Multiple missing references noted

**Answer: The missing references were inserted**.

7. Typographical error in limitations “a large number many patients”

**Answer: The typo was corrected**

Impression - Overall a disappointing discussion lacking actual true analysis and speculation on the results found. A noticeable part of it was discussing the studies against the findings for orthostatic hypotension and other parts not fully described e.g. the biopsies done. There was no acceptable differentiation of the different phenotypes of where they differed. It appears the data was collected, not properly presented or explained and just laid out instead of interpreting the data to explain what differences, similarities between the phenotypes and how it relates to a clinical picture. Much of the focus remains on trying to convince the reader the similarities without actually explaining the data. It was striking how little was relayed about the results of the patients and consistently comparing with the different groups especially the healthy control group as I believe they were almost never mentioned. Much more details and deliberation would be needed for this study to make clinical sense as currently it is poorly detailed statistical analysis to detect certain anomalies in these patients then trying to find similarities without having the data itself show the similarities and differences.

Answer: We only could discuss the significant findings. We were surprised how our findings are nonspecific as they are also present in hEDS group.

In addition, lack of blood markers

When submitting your revision, we need you to address these additional requirements.

1. Please ensure that your manuscript meets PLOS ONE's style requirements, including those for file naming. The PLOS ONE style templates can be found at

<https://journals.plos.org/plosone/s/file?id=wjVg/PLOSOne_formatting_sample_main_body.pdf> and

[https://journals.plos.org/plosone/s/file?id=ba62/PLOSOne_formatting_sample_title_authors_affiliations.pdf](https://secure-web.cisco.com/1qORP7XGLKEOnmitDgGHmCjex5psWUZL4fUM-Qiqcfqnpxf1URwBDd_l__GDEOOT2VjMv95ZsROU-t6bcvfWWNE8KupK5Z2r0VLpPNoab0_22wkXEYHBvytLvw3PzvWUyzmHmn00YLOz-A1PrJld91UxH7bqiH2EjOa7RDiUMbdGn6trLffWRdudqvWcW0HXaCg91B1KL41_NwNPzuAKOyeb7hxa49AL3uGudMXwgcVR2M4f0xyXcxSt8XmJBTphPGYKo6hNoy1dbXzsno0Eg9ChLIyIsKkZMT86jBbxwsA-GyL2pivT5vn3koiZA9b2R/https%3A%2F%2Ftrack.editorialmanager.com%2FCL0%2Fhttps%3A%252F%252Fjournals.plos.org%252Fplosone%252Fs%252Ffile%253Fid%3Dba62%252FPLOSOne_formatting_sample_title_authors_affiliations.pdf%2F1%2F010f0197c5e0e317-6abb83b0-8682-4ab1-b143-a52566126a13-000000%2FLAR_JRQGG3qM_rDM4_cXsd2ilw8lwIAOxcuFY-665VU%3D218)

**Answer: The manuscript was reformatted to be able to meet the PLOS ONE style.**

2. Please expand the acronym “FBRI” (as indicated in your financial disclosure) so that it states the name of your funders in full.
This information should be included in your cover letter; we will change the online submission form on your behalf.

**Answer: The full name of the sponsor is FBRI LLC**

https://seniornavigator.org/program/76390/grant-making-organization-alzheimers-disease

3. Thank you for stating the following financial disclosure:
This work was funded by Mona Taliaferro/Bay Shore Recycling, The National Heart, Lung and Blood Institute (NHLNI - 1OT2HL156812-01) and FBRI (2022A018462) to P. Novak.

Please state what role the funders took in the study. If the funders had no role, please state: "The funders had no role in study design, data collection and analysis, decision to publish, or preparation of the manuscript."
If this statement is not correct you must amend it as needed.
Please include this amended Role of Funder statement in your cover letter; we will change the online submission form on your behalf.

**Answer: The role of funders was included to the manuscript as well as to the cover letter.**

4. In the online submission form, you indicated that restricted data will be available from the corresponding author upon reasonable request.

All PLOS journals now require all data underlying the findings described in their manuscript to be freely available to other researchers, either a. In a public repository, b. Within the manuscript itself, or c. Uploaded as supplementary information.
This policy applies to all data except where public deposition would breach compliance with the protocol approved by your research ethics board. If your data cannot be made publicly available for ethical or legal reasons (e.g., public availability would compromise patient privacy), please explain your reasons on resubmission and your exemption request will be escalated for approval.

**Answer: The de-identified data will be available on reasonable request approved by ethic board.**

5. Please amend your list of authors on the manuscript to ensure that each author is linked to an affiliation. Authors’ affiliations should reflect the institution where the work was done (if authors moved subsequently, you can also list the new affiliation stating “current affiliation:….” as necessary).

**Answer: List of authors was amended to reflect correct affiliations**

6. Please include your tables as part of your main manuscript and remove the individual files. Please note that supplementary tables (should remain/ be uploaded) as separate "supporting information" files

**Answer: The tables were attached to the main manuscript.**

7. Please include captions for your Supporting Information files at the end of your manuscript, and update any in-text citations to match accordingly. Please see our Supporting Information guidelines for more information: <http://journals.plos.org/plosone/s/supporting-information>.

**Answer: The captions were inserted in the main manuscript.**

8. Please remove all personal information, ensure that the data shared are in accordance with participant consent, and re-upload a fully anonymized data set.

Note: spreadsheet columns with personal information must be removed and not hidden as all hidden columns will appear in the published file.

**Answer: all personal information was removed.**

Additional guidance on preparing raw data for publication can be found in our Data Policy (<https://journals.plos.org/plosone/s/data-availability#loc-human-research-participant-data-and-other-sensitive-data>) and in the following article: [http://www.bmj.com/content/340/bmj.c181.long](http://secure-web.cisco.com/18EL-HGE2JamdGy88git-yyIdxBHEd4gRQY24fpxaiyWVTVYSl-LC9lefl9jM4k9a9tZB3Wu4YbX8KJ9PousTwDtimuz9wQzj8uad9z0tYBVjvjaoIskpRkT8_TI4FeUgAzFNBP-kNkfcVr2-NaSABV3Ffr4NmX3Ed_mIO9HxR9Xqlcy4fVE0c2G-SoLWjqHStaZwh-avKifD7HtMtFkiAI-8pOCi-cIhJrTgP-MgFHN3dGJEeRM4zB_gJo-dUsZlK4FCwQozzMMXZZwfHZ4F1usvU7vT_IYGJTgGtSIfQ26U9VoUUudhxtavNu2Glfpx/http%3A%2F%2Ftrack.editorialmanager.com%2FCL0%2Fhttp%3A%252F%252Fwww.bmj.com%252Fcontent%252F340%252Fbmj.c181.long%2F1%2F010f0197c5e0e317-6abb83b0-8682-4ab1-b143-a52566126a13-000000%2Fp1HFmAKLQHdg2vVWwsiMvoNocol3paMz8TK-9p5OZP0%3D218).

5. Review Comments to the Author

Please use the space provided to explain your answers to the questions above. You may also include additional comments for the author, including concerns about dual publication, research ethics, or publication ethics. (Please upload your review as an attachment if it exceeds 20,000 characters)

Reviewer #1: Your manuscript requires major revisions to improve clarity, structure, and the robustness of your analysis. The introduction lacks sufficient background information and references, making it difficult for readers unfamiliar with the topic to follow. The methods section needs better organization, clearer inclusion/exclusion criteria, and a more readable presentation of diagnostic tools like QASAT. The statistical analysis is incomplete—key details such as power calculations, handling of outliers, and confounder adjustments are missing, undermining confidence in the results. The results section is disorganized, presenting random patient factors without context, making interpretation difficult. The discussion lacks meaningful analysis of the data, failing to draw strong conclusions or explore clinical implications. Key recommendations: improve readability, ensure statistical rigor, clearly present patient characteristics, provide a structured interpretation of findings, and avoid excessive acronyms.

Reviewer #2: This manuscript studied the shared autonomic phenotype of Long COVID and ME/CFS, which is very interesting and important in post COVID era. The manuscript is beautifully written, and I truly enjoyed reading through the paper, in which the main conclusions are very well supported by the data presented. The authors have done an excellent job. Meaning while, I have few minor comments:

1. Figure resolution need be improved.

**Answer: If the paper will be accepted, we can provide a figures in vector format, that has much higher resolution.**

2.Data point should be showed in the figures, box plots and violin plots are preferred.

**Answer: We created the violin plots, they look very confusing, therefore we prefer the bar plots.**
3. For long COVID patients, did the author considered the infection times? This should be discussed.

**Answer: We did collect the infection times. Due to the small number of pre-Omicron cases, we did not perform a comparative analysis of the effects of different SARS-CoV-2 strains. This is mentioned in the discussion.**

4. Why the authors choose ME/CFS for the comparation? More detailed explanation in the introduction section would be helpful.

**Answer: The reason for Long COVID and ME/CFS comparison is the theory that both conditions share similar pathophysiology, that is mentioned in the introduction. We added more details about these similarities.**

**In addition, we made number of other updates to improve the manuscripts.**
