# Supplementary material for: Shared autonomic phenotype of long COVID and myalgic encephalomyelitis/chronic fatigue syndrome
Source: PLoS One. 2026 Jan 23;21(1):e0341278. doi: 10.1371/journal.pone.0341278 (PMC12829881; doi:10.1371/journal.pone.0341278)
Supplement: Attachment — Submitted filename: Response to Reviewers.docx [file pone.0341278.s003.docx]

Dear Editor

We would like to express our sincere gratitude to both reviewers for their constructive feedback. We have carefully revised the paper in accordance with the suggestions. Below, we provide detailed responses to each of the reviewers' comments:

For authors

Peter Novak

Reviewer #1: Much morei improved, legible and able to discern the quality points made by the authors. There are a few grammatical errors noted eg. Page 22 "di not confirm this finding

I would recommend adding quantifying data when comparing how simliar or different the disorders are in certain test. For example it was noted central cerebrovascular flow was more diminished in COVID patients vs hED. Adding by how much etc. I find the discussion was lacking the actual quantifying data

Also recommend adding smaller tables that shows the highlighted points in the tests with the concurrent P Values and Confidence intervals rather than relying on the large table at the end.

**Answer:** Thank you for your suggestions. We added the Table 6 that summarizes the differences between Long COVID and ME/CFS. In the table 6 we focus in comparisons between Long COVID and ME/CFS because that was the main topic of the study. For that reasons we did not include Controls and hEDS in the table 6. We also added the difference in the discussion, for example to the sentence:

Both our patient groups exceeded that level of decline.

was expanded to:

Both our patient groups exceeded that level of decline (Long COVID –25% and ME/CFS -22%).

The sentence:

Our study detected frequent autonomic failure in both Long COVID and ME/CFS .

Was expanded to:

Our study detected frequent autonomic failure in both Long COVID (95%) and ME/CFS (89%).

For consistency, we prefer to use the sd (standard deviations) and not confidence intervals because we use sd’s in all other tables. Both sd’s and confidence intervals, together with p values are good measures of data variability.

We also added the paragraph discussing the central sensitization since we believe, this is important finding, particularly in Long COVID patients.

In addition, we corrected several typos and changed legends in figures (PASC was replaced with Long COVID).
